# Interaction of the dorsolateral prefrontal cortex with the precuneal medial parietal cortex for the monitoring of information in working memory in the macaque monkey

**DOI:** 10.1093/cercor/bhae315

**Published:** 2024-08-07

**Authors:** Michael Petrides, Sarah Lefebvre, Jennifer Novek, Veronika Zlatkina

**Affiliations:** Cognitive Neuroscience Unit, Montreal Neurological Institute, McGill University, 3801 University St, Montreal, Quebec H3A 2B4, Canada; Cognitive Neuroscience Unit, Montreal Neurological Institute, McGill University, 3801 University St, Montreal, Quebec H3A 2B4, Canada; Cognitive Neuroscience Unit, Montreal Neurological Institute, McGill University, 3801 University St, Montreal, Quebec H3A 2B4, Canada; Cognitive Neuroscience Unit, Montreal Neurological Institute, McGill University, 3801 University St, Montreal, Quebec H3A 2B4, Canada; Rogue Research Inc., 6666 Saint Urbain St, Montreal Quebec H2S 3H1, Canada

**Keywords:** attentional network, area 46 and area 9/46, area PGm, executive control processing, precuneus

## Abstract

The executive control process of monitoring information in working memory depends on the mid-dorsolateral prefrontal cortical region (cytoarchitectonic areas 46 and 9/46) in interaction with the hippocampal memory system. Anatomical studies demonstrated strong connectivity between the mid-dorsolateral prefrontal cortex and the medial parietal area PGm that lies on the precuneus. Area PGm is also strongly connected with the attentional system on the lateral inferior parietal lobule (area PG) and the limbic retrosplenial/posterior cingulate region that interacts with the hippocampal memory system. Thus, in terms of anatomical connectivity, area PGm appears to be a critical node for the integration of executive control processing from the prefrontal cortex with the online attentional and memory related processing. This hypothesis was tested in macaque monkeys with the crossed unilateral lesion methodology. A unilateral lesion in the mid-dorsolateral prefrontal cortex was combined with a unilateral lesion in area PGm in the opposite hemisphere. The results demonstrated an impairment on the externally ordered working memory task that assesses the monitoring of information in working memory. Thus, the medial parietal area PGm is a critical node in mediating the functional interaction between the prefrontal region for the executive control process of monitoring information and the memory system.

## Introduction

**Fig. 1:**
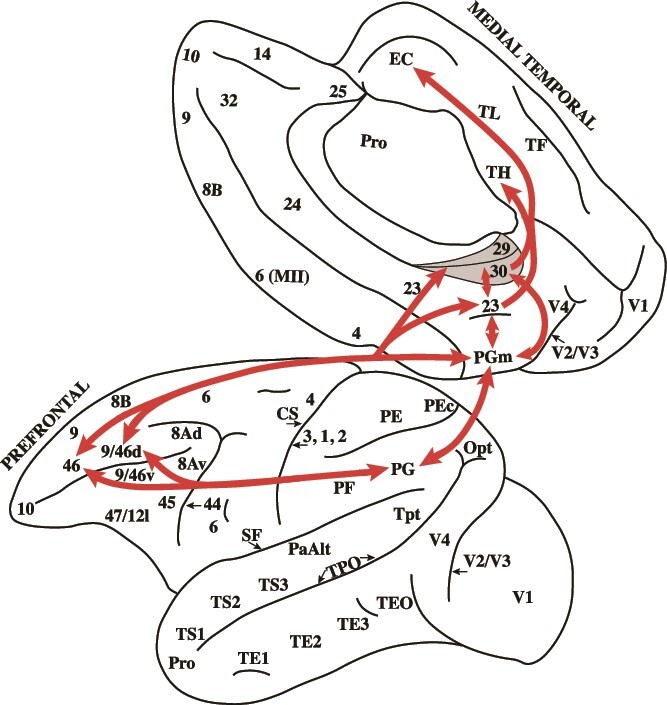
The lateral and medial surfaces of the macaque monkey hemisphere. The arrows show the connectivity between the relevant cortical regions. Note that medial parietal area PGm is bi-directionally connected with the mid-dorsolateral prefrontal region (areas 46 and 9/46), area PG on the lateral inferior parietal lobule, and the retrosplenial cortex within the splenial sulcus (areas 29 and 30) and the posterior cingulate gyrus (area 23), i.e. the retrosplenial/posterior cingulate region. Note that the retrosplenial/posterior cingulate region is strongly connected with the hippocampal memory system on the medial temporal region, i.e. the entorhinal cortex (EC) and area TH. Thus, anatomically, the medial parietal area PGm on the precuneus appears to be a critical node for integrating information from the mid-dorsolateral prefrontal cortex and lateral parietal cortex and providing access to the hippocampal memory system.

The mid-dorsolateral prefrontal cortex (cytoarchitectonic areas 46 and 9/46) is the critical prefrontal region for the executive control process of monitoring information in working memory (Petrides 1991, 1995, 2000) in functional interaction with the hippocampal memory system (Petrides and Milner 1982). Note, however, that there are no direct connections of the mid-dorsolateral prefrontal cortex with the hippocampus. The mid-dorsolateral prefrontal region is strongly connected with the inferior parietal lobule (area PG) on the lateral surface of the hemisphere and the medial parietal area PGm on the precuneus (Petrides and Pandya 1999). There is considerable research demonstrating that the executive control process of monitoring by the mid-dorsolateral prefrontal cortex interacts with the inferior parietal cortex (area PG), which is a central hub providing the attentional context within which multisensory events are processed (Behrmann et al. 2004; Shomstein and Gottlieb 2016). This fronto-parietal network is the critical online network for monitoring information within working memory (see Petrides 2013 for review). How does this online fronto-parietal network interact with the hippocampal memory system? Note that the precuneal medial parietal area PGm is strongly connected both with the mid-dorsolateral prefrontal cortex and the inferior parietal cortex (area PG) on the lateral surface of the hemisphere and the ventrally adjacent limbic retrosplenial/posterior cingulate region that provides access to the hippocampus (Pandya and Seltzer 1982; Leichnetz 2001) (see Fig. 1). Thus, based on the anatomical connectivity, the medial parietal area PGm on the precuneus appears to be a critical site for integrating executive control from the mid-dorsolateral prefrontal cortex with the online multisensory attentional processing in the inferior parietal cortex (area PG) and providing access to the hippocampal memory system. Note that, in the human brain, the medial parietal cortical area PGm was labeled as medial area 7 in the Brodmann cytoarchitectonic map and as medial area PE in the Economo and Koskinas map (see Brodmann 1909; Economo and Koskinas 1925).

In the examination of the cytoarchitecture of the posterior parietal cortex in the macaque by Pandya and Seltzer, the medial parietal cortical region on the precuneus was labeled as area PGm to emphasize (i) the similarity of its cytoarchitecture with that of the lateral inferior parietal lobule area PG and (ii) the strong bi-directional connectivity between these two posterior parietal cortical areas (Pandya and Seltzer 1982).

Margulies et al. examined with resting-state functional magnetic resonance imaging the connectivity of the precuneal region in the human brain and the results were consistent with the macaque monkey neuroanatomical evidence described above (Margulies et al. 2009). The central precuneal region (where medial parietal area PGm lies) was functionally connected with the dorsolateral prefrontal cortex, the multimodal lateral inferior parietal cortex area PG (also known as Brodmann area 39 in the human brain), and the ventrally adjacent limbic retrosplenial/posterior cingulate gyrus that is connected with the medial temporal region where the hippocampal memory system lies. Thus, in the human brain, also, the functional connectivity of the precuneal region (i.e. medial parietal area PGm) suggests that it may be a critical node between the executive control emanating from the dorsolateral prefrontal cortex, the multisensory inferior parietal lobule attentional system and the memory related region in the retrosplenial/posterior cingulate gyrus. Furthermore, in functional neuroimaging studies examining activation during the monitoring of information in working memory tasks, in addition to the dorsolateral prefrontal cortex and the lateral parietal cortex (i.e. the fronto-parietal network), there was activation in the precuneal medial parietal area PGm, i.e. area 7 in the Brodmann terminology (Petrides et al. 1993a, 1993b). Thus, both resting state connectivity and functional activation studies support the idea that medial parietal area PGm may be a critical node for the integration of information from dorsolateral prefrontal and lateral parietal cortex and providing access to the hippocampal memory system.

**Fig. 2:**
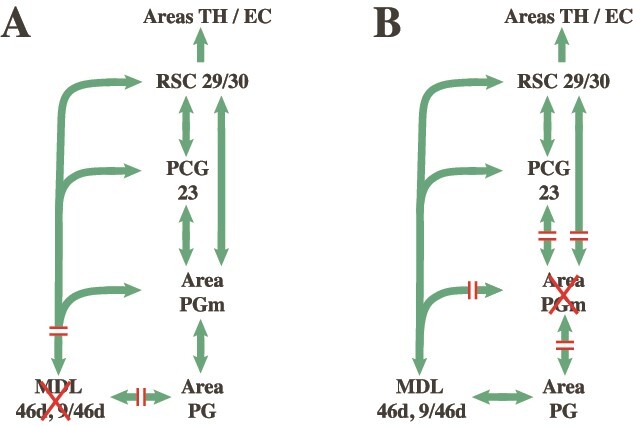
Schematic illustration of connections (arrows) and disconnections (**//**) produced by a lesion (A) to the MDL (areas 46d and 9/46d) in one hemisphere and (B) a lesion to area PGm on the precuneus in the contralateral hemisphere. Abbreviations: MDL, mid-dorsolateral prefrontal region (areas 46d, 9/46d); PCG, posterior cingulate gyrus (i.e. area 23); RSC, retrosplenial region (i.e. areas 29 and 30); TH/EC, area TH and entorhinal cortex (EC) on the medial parahippocampal gyrus.

The present investigation examined the above hypothesis about the role of the precuneal medial parietal region (area PGm) during the monitoring of information in working memory. Bilateral brain lesions to an area, such as the mid-dorsolateral prefrontal cortex, demonstrate the critical functional role of that area, such as its role in the monitoring of information in working memory (Petrides 1991, 1995, 2000), but fail to provide evidence of its functional interaction with other brain areas, such as the medial parietal region (area PGm). Evidence for functional interaction between cortical areas regarding a specific cognitive process is classically demonstrated by the crossed unilateral lesion method, i.e. making a unilateral lesion in area A in one hemisphere and a unilateral lesion in area B in the opposite hemisphere (see Vaidya et al. 2019, for discussion of this method). Thus, a unilateral lesion in the mid-dorsolateral prefrontal cortex in one hemisphere (e.g. left hemisphere) would allow normal functioning of this area in the opposite right hemisphere, but the unilateral lesion in the medial posterior parietal region (PGm) in the right hemisphere would prevent the normal interaction of the intact mid-dorsolateral prefrontal cortex with cortical areas linked via area PGm, such as the ventrally adjacent limbic retrosplenial/posterior cingulate region that influences the hippocampal memory system (see Fig. 2).

As pointed out above, anatomically, the precuneal medial parietal area PGm appears to be a critical node permitting the interaction between the executive control processing in the prefrontal cortex, the attentional system in the lateral posterior parietal region (area PG) and the retrosplenial/posterior cingulate region which provides access to the hippocampal memory system. Thus, a lesion in the mid-dorsolateral prefrontal cortex (areas 46 and 9/46) in one hemisphere and a contralateral lesion of medial parietal area PGm in the opposite hemisphere will anatomically disconnect the interaction of the mid-dorsolateral prefrontal executive control process of monitoring information within working memory from the hippocampal memory system (see Figs. 1 and 2). This is the hypothesis that was tested in the present experiment. The macaque monkeys were trained preoperatively and tested postoperatively on a task requiring the monitoring of information in working memory, the Externally Ordered Monitoring (EOM) working memory task that has been used reliably to assess the role of the mid-dorsolateral prefrontal cortex in monitoring information within working memory (Petrides 1995). The decision to use a nonspatial version of the EOM task was made so that the role of these areas would be shown to underlie the general processing of information in working memory.

## Materials and methods

### Subjects and surgical procedure

The subjects were 1 female and 2 male adult monkeys (*Macaca fascicularis*). Two of the monkeys (1 female, F1, and 1 male, M1) were operated on, and 1 male monkey was the non-operated control (NC) animal. Macaques F1 and M1 received crossed unilateral lesions of the mid-dorsolateral prefrontal cortex (where areas 46 and 9/46 lie) in one hemisphere (F1: left hemisphere; M1: right hemisphere) and the medial parietal cortical region (where area PGm is located) in the other hemisphere. The mid-dorsolateral prefrontal cortical lesions were intended to extend above the middle sector of the sulcus principalis, sparing the most rostra1 part of the lateral frontal cortex, i.e. the frontopolar area 10. In making the mid-dorsolateral cortical lesions, great care was taken not to damage or undercut the connections of the inferior frontal convexity, that is, the ventrolateral frontal cortex that lies below the sulcus principalis. The medial parietal cortical lesion was intended to remove the central part of the precuneus, i.e. area PGm. The surgical operations in the brains of the 2 macaque monkeys, F1 and M1, were performed after the preoperative training (see below) and the monkeys were re-tested postoperatively after a recovery period of 2.5 weeks. The normal control monkey (NC) received the same preoperative training and was re-tested after a 6 week period.

All surgical procedures were carried out under strict aseptic conditions and followed the standard operating procedures of McGill University for non-human primate analgesia, anesthesia, surgery, preoperative and postoperative care. The experiments were performed in accordance with a protocol approved by McGill University and the Montreal Neurological Institute-Hospital (The Neuro) Animal Care Committees, in accordance with guidelines established by the Canadian Council on Animal Care. The surgery was carried out by standard aseptic operating procedures for the subpial aspiration of cortical tissue, i.e. ablation of a small focal part of the cortex.

### Histological procedure

At the completion of the experiment, the 2 operated animals were deeply anesthetized with sodium pentobarbital (100 mg/kg, I.V., Euthanyl, Bimed-MTC animal health, Canada) and perfused transcardially with heparinized saline, followed by a solution of 4% paraformaldehyde. The brains of the animals were removed and fixed in a 4% paraformaldehyde solution. The two hemispheres of each brain were separated along the corpus callosum and each hemisphere was divided into blocks. Digital photographs were taken of the hemispheres and the individual blocks of tissue. For cryoprotection, the blocks were immersed in 10, 20, and 30% solutions of sucrose until they were fully submerged. The frontal block that contained the mid-dorsolateral prefrontal cortical region and the parieto-occipital block that contained the medial parietal cortical region were frozen and sectioned coronally at 30 μm thickness using a sliding microtome. Every 10th histological section was stained with cresyl violet (Nissl stain). A microscopic examination of the stained sections was conducted and drawings of the lesions were made.

### Behavioral testing

The monkeys had constant access to water in their home cages (i.e. they were not on water deprivation). The animals were tested daily in the morning for 30 to 45 min. After the testing, the animals received in their home cages their daily food intake in the form of dry non-human primate food pellets, as well as fruits and vegetables. Thus, the next morning they were motivated for their behavioral testing for food pellets and fruit. Their daily food consumption and weight were kept on record. The animals were tested for 5 days during each week, and were rewarded with dried fruit (e.g. raisins, dried papaya) and nuts (e.g. almonds, peanuts) for correct responses. These rewards were tailored to each animal’s personal preference.

Testing was carried out in a Wisconsin General Testing Apparatus which consists of a compartment where the monkey is held and a testing area. The compartment where the monkey is located is separated from the testing area by an opaque screen that can be opened to give access to the testing area and closed to occlude the monkey’s view of the testing area. The visual stimuli used in this study were common household objects, such as mugs, penny banks, pen holders, metal decorative boxes, building blocks, decorative items, etc.

### Preoperative testing

The aim of the present experiment was to examine the effect of the crossed unilateral lesions within the mid-dorsolateral prefrontal cortex (areas 46 and 9/46) in one hemisphere and the medial parietal cortical region (area PGm) in the other hemisphere on the ability of macaques to monitor information within working memory. Before training on the nonspatial working memory task, the animals received training on visual discrimination tasks so that they would learn to focus their attention on the visual characteristics of the stimuli and ignore their location, as well as to learn that certain selections are the correct responses and are thus rewarded and other responses are incorrect and are not rewarded.

In the visual discrimination tasks, a pair of objects was presented across all testing trials and one of the stimuli was always the rewarded stimulus and the other stimulus was never rewarded. Thus, the monkeys had to learn to attend to and select the rewarded stimulus and ignore the unrewarded stimulus. After reaching criterion on a particular visual discrimination task, the reward relations were reversed, i.e. the previously rewarded stimulus was now the negative unrewarded stimulus and the previously unrewarded stimulus was now rewarded. On completion of the training on the visual discrimination and reversal tasks, the animals were tested on the classical delayed non-match to sample task. In this visual recognition memory task, the animal is presented with one stimulus on the presentation trial and, after a delay, that stimulus is paired with a new stimulus and the animal must select the new stimulus. Thus, the decision here is to recall the previously presented stimulus and not to re-select it after the delay interval. Following the preliminary training on the visual discrimination and delayed non-match to sample tasks, the monkeys were trained on the EOM working memory task that assesses the monitoring of information in working memory (Petrides 1995).

### EOM working memory task

**Fig. 3:**
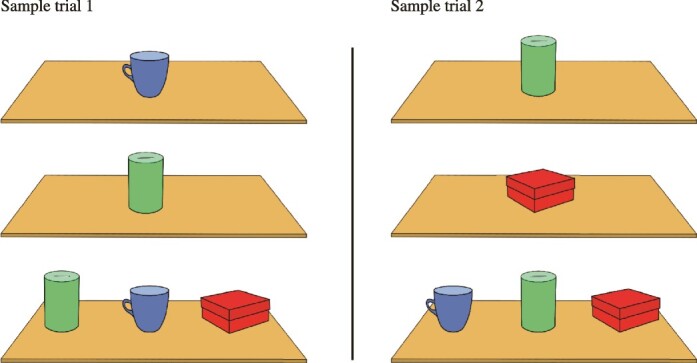
Schematic illustration of the EOM-3. The same objects are used in all trials. A subset of the objects (randomly chosen) is shown sequentially on the first and second presentation phases. On the test phase of the trial that follows, the monkey sees all 3 objects arranged in a random sequence and must select the object that was not shown on the presentation phases of the trial. Thus, the monkey must monitor the occurrence/non-occurrence of the stimuli across time (working memory) to be able to select correctly the non-presented item on the test phase of the trial. Note that the same stimuli are used in all trials. Two sample trials are shown in the illustration.

In the EOM-3 working memory task, the *same* 3 objects were used in all trials (see Fig. 3). The test board had 3 wells, 10 cm apart, over which the stimulus objects were presented. Each trial had three phases: the first presentation phase during which 1 of the 3 objects was randomly selected and presented over the middle well, then the second presentation phase during which 1 of the 2 remaining objects was randomly selected and presented again over the middle well and, finally, the test phase during which all 3 objects were presented in a row in a random arrangement according to the Gellermann schedule (Gellermann 1933). The monkey now had to select the object that had not been presented on the two presentation phases of the trial to receive reward.

When the opaque screen was raised in the first presentation phase of a trial, the animal saw 1 of the 3 objects in the middle of the test board (Fig. 3) and was required to touch the object. The screen was then lowered, the object was replaced with another one in the middle of the test board, and, after a delay of 7 to 10 s, the opaque screen was raised again to allow the monkey to touch the object in the second presentation phase of the trial. The screen was lowered once more, and, when it was raised, after the delay of 7 to 10 s, the testing phase of the trial was administered. The animal was now faced with all 3 objects placed in a row on the test board. Only the object that had not been shown on the previous presentation phases of the trial had a reward under it, and, if the animal responded correctly by displacing that object, the animal retrieved the reward and that particular trial was terminated. If the animal made an error by displacing 1 of the 2 objects that were presented on the first two presentation phases of the trial, the screen was lowered, and a new trial was administered. Note that the 2 objects for the presentation phases of the next trial were randomly selected from the set of the same 3 objects. The criterion of successful performance was 18 correct trials out of 27 daily trials (67%) for 2 consecutive days. The last 20 days of training on the task was considered the preoperative performance of the animals and was used to make comparison with the postoperative performance.

After the monkeys had completed training on the EOM-3 task, they were operated. Postoperatively, after the recovery period (see above), the monkeys were tested on the EOM working memory task with the *same* 3 stimuli (EOM-3) for 20 days. In addition, postoperatively, the 2 monkeys with lesions were tested on the EOM working memory task with 4 stimuli (EOM-4) for 10 days. The structure of the EOM working memory task with 4 stimuli was the same as that with the 3 stimuli. Four objects were used for this version of the task, and these objects were used repeatedly in all trials. The 4 objects were the same as those in the EOM-3 with an additional object. Each trial now had three presentation phases during which 3 of the 4 objects were randomly selected and were presented in sequence and, in the test phase of the trial, all 4 stimuli were presented in a row and the monkeys were required to select the 1 of the 4 objects that had not been presented on the three presentation phases of that particular trial.

Statistical comparisons were carried on the postoperative performance of the monkeys on the EOM-3 and EOM-4 tasks using t-tests. Specifically, on the EOM-3 task, we compared the postoperative performance of monkeys M1, F1, and NC. On the EOM-4, the postoperative performance of monkey M1 was compared to the performance of monkey F1. In addition, we compared the postoperative performance of monkey F1 during the first 10 days of testing on the EOM-3 task to the 10 days of testing on the EOM-4 task, and the same comparison was carried out for monkey M1. The performance of monkeys M1 and F1 on the EOM-4 task was also compared to chance performance on this task which is 25%.

## Results

### Description of the lesions

**Fig. 4:**
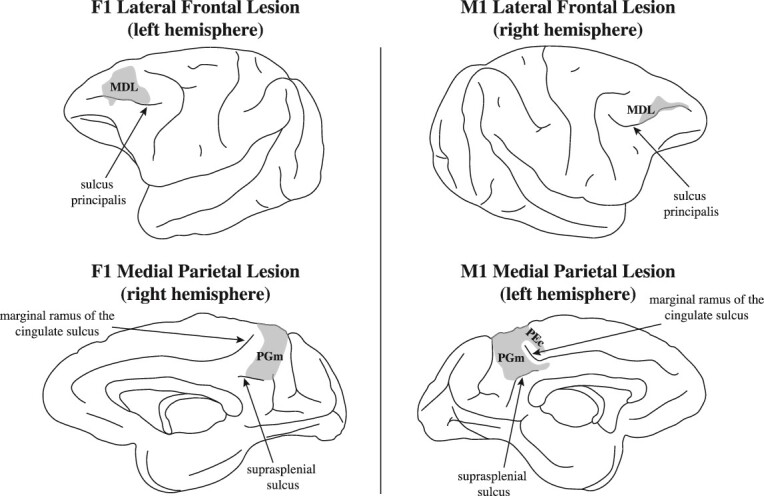
Location of crossed unilateral lesions in macaque monkeys F1 and M1. Note that the frontal lesion in monkey F1 involves the entire mid-dorsolateral prefrontal region (areas 46 and 9/46). The frontal lesion in monkey M1 is restricted to the anterior part of the mid-dorsolateral prefrontal region, sparing its posterior part. Note that monkey F1 demonstrated the more severe impairment (see Fig. 5). On the medial hemisphere of monkey F1, the lesion was restricted to the precuneal medial parietal area PGm. On the medial hemisphere of monkey M1, the lesion included area PGm, but extended above the marginal ramus of the cingulate sulcus to include a small portion of area PEc. Abbreviations: MDL, mid-dorsolateral prefrontal region; area PGm on the medial parietal cortical region, i.e. the precuneus.

A microscopic examination of the Nissl stained sections of the brains of the 2 operated monkeys was conducted and drawings of the lesions were made (see Fig. 4). The microscopic examination confirmed that, in the lesions, the gray matter was removed as intended with minimal involvement of the underlying white matter. In macaque monkey F1, there was a lesion of the mid-dorsolateral prefrontal cortex in the left hemisphere. The lesion involved the upper bank of the middle third of the sulcus principalis where the dorsal parts of areas 46 and 9/46 lie. In the right hemisphere of monkey F1, the lesion was in the medial parietal cortex limited to precuneal area PGm. The ventral limit of the lesion was the suprasplenial sulcus and it extended dorsally to the midline (see Fig. 4). Note that there was no damage to the cortex anterior to area PGm, i.e. the cortex within and just posterior to the marginal ramus of the cingulate sulcus (i.e. area PEc). Note also that the retrosplenial cortex (areas 29 and 30) within the splenial sulcus just above the splenium of the corpus callosum and the posterior cingulate gyrus (area 23) were intact. In macaque monkey M1, the frontal lesion was placed in the right hemisphere within the upper bank of the sulcus principalis and the immediately adjacent dorsal region. The lesion in this monkey involved primarily dorsal area 46, and the dorsal area 9/46 was also partially affected (see Fig. 4). The contralateral lesion was placed within the left hemisphere in the medial precuneal region. The lesion involved the middle part of the precuneal cortex (i.e. area PGm), but also extended rostrally to involve the cortex dorsal to the marginal ramus of the cingulate sulcus. Thus, there was some damage to area PEc.

### Postoperative behavioral performance

**Fig. 5:**
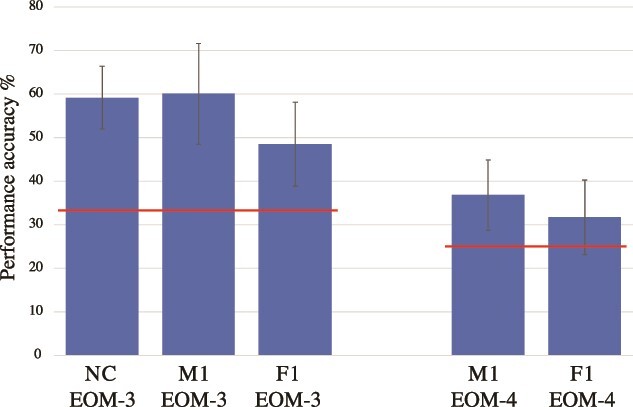
Postoperative performance in the EOM working memory task. The figure shows the mean percent performance and the standard deviation. The horizontal (red) lines indicate the chance probability (33%) in the EOM-3 task and the chance probability (25%) in the EOM-4 task. Abbreviations: EOM-3, Externally Ordered Monitoring working memory task with 3 stimuli; EOM-4, Externally Ordered Monitoring working memory task with 4 stimuli; F1 and M1, operated monkeys; NC, normal control monkey.

Preoperatively, the monkeys were trained on the EOM task that assesses the executive process of monitoring information in working memory (Petrides 1995). In this task, the monkey sees on every trial, in sequence, a random selection from the same set of stimuli and must keep track (i.e. monitor in memory) those stimuli that were presented as opposed to the stimulus that was not presented on each particular trial. For example, in a 3 object task, 2 of the 3 stimuli (randomly selected) are presented in sequence and, on the test phase of the trial, the monkey must select the one object that was not presented on that trial. Once the monkeys had reached the preoperative criterion of successful performance on the EOM-3 task, they were operated. The 3 monkeys were tested postoperatively on the EOM task with 3 objects (EOM-3) and the 2 operated monkeys were also tested with 4 objects (EOM-4). On the EOM-3, the unoperated control monkey (NC) and M1 performed at the same level, but F1 performed lower than the NC and M1 and was, therefore, impaired. A comparison of the postoperative performance of M1 with that of the unoperated control monkey was not significant (t(19) = 0.2965, *P* > 0.05). By contrast, the postoperative performance of macaque F1 differed significantly from the control macaque (t(19) = 3.9802, *P* < 0.01) and macaque M1 (t(19) = 3.4577, *P* < 0.01) (see Fig. 5).

On the EOM task with 4 objects (EOM-4), the performance of macaques F1 and M1, who had the crossed unilateral lesions, did not differ significantly from each other (t(9) = 1.3363, *P* > 0.05) and the performance of both monkeys was significantly lower than their performance on the 3 object EOM task (see Fig. 5). Specifically, in this comparison the first 10 days of postoperative testing on the EOM-3 task were compared with the 10 days of postoperative testing on the EOM-4 task in monkey M1 (t(9) = 4.4978, *P* < 0.05) and monkey F1 (t(9) = 3.2448, *P* < 0.05). Note that monkey M1 who was not impaired on the EOM-3 task was performing on the EOM-4 task at the level of monkey F1 who was impaired on the EOM-3 task. Because the one NC monkey in this experiment was not available for testing on the EOM-4 task, we should mention that previous testing of normal control monkeys on working memory monitoring tasks with 4 and 5 stimuli yielded correct performance at over 70% (e.g. Petrides 2000). On the EOM-4 task, the performance of macaque M1 (t(9) = 4.671, *P* < 0.05) and macaque F1 (t(9) = 2.5733, *P* < 0.05) was significantly different from the chance performance of 25%.

Monkey F1 had a larger lesion in the mid-dorsolateral prefrontal cortex compared with monkey M1 and the crossed lesion in the posterior medial cortex was restricted to the target area, i.e. PGm. Thus, there was a clearer disconnection in this macaque monkey between the two critical regions, i.e. the mid-dorsolateral prefrontal cortex and medial parietal cortex. Monkey M1 had a smaller lesion in the mid-dorsolateral prefrontal cortex (see Fig. 4), although the lesion in the medial parietal cortical region was larger. The smaller lesion in the mid-dorsolateral prefrontal cortex of this monkey may explain the lack of impaired performance on the easier version of the EOM, i.e. the 3 stimulus task. On the EOM task with 4 objects, monkey M1 performed at the same impaired level as the monkey F1 who had the more complete disconnection between the two critical regions. Thus, the data are consistent with the interpretation that medial parietal area PGm is a *critical* node permitting functional interaction between the mid-dorsolateral prefrontal cortex (areas 46 and 9/46), the lateral inferior parietal lobule (area PG), and the retrosplenial/posterior cingulate region that provides access to the hippocampal memory system for the monitoring of information within working memory (see Figs. 1 and 2).

Note that, in earlier studies, we found that bilateral lesions of the mid-dorsolateral prefrontal cortex (areas 46 and 9/46) impaired performance on the working memory monitoring tasks, but bilateral lesions of prefrontal area 8A and dorsal area 6 (which are just posterior to the critical mid-dorsolateral prefrontal cortex) did not impair performance on the monitoring tasks (Petrides 1991, 1995, 2000). The lack of impairment after bilateral lesions of area 8A and dorsal area 6 suggests that a “mass action” effect (i.e. extent of cortical excision) does not explain the observed impairment. In the present experiment, it is the disturbance of the functional interaction between the mid-dorsolateral prefrontal cortex in one hemisphere and the medial parietal area PGm in the other hemisphere that explains the impaired performance. This is the power of the crossed unilateral lesion methodology that is used to examine functional interactions between areas (see, Vaidya et al. 2019).

## Discussion

The mid-dorsolateral prefrontal cortex (areas 46 and 9/46) in interaction with the multisensory attentional system in the inferior parietal lobule (area PG) constitutes the fronto-parietal network for the *online* monitoring of information (Petrides 2013). For the monitoring of information across time (i.e. involving memory), this fronto-parietal network must interact with the hippocampal memory system (Petrides and Milner 1982). The present study examined the functional effect of disconnecting this fronto-parietal network for the online monitoring of information from the hippocampal memory system via a cortical excision of the medial parietal area PGm on the precuneus. The medial parietal area PGm has the necessary anatomical connectivity to integrate information from the mid-dorsolateral prefrontal and inferior parietal attention network for the online monitoring of information with the memory system via the limbic retrosplenial/posterior cingulate cortex that provides major input to the hippocampal system (see Figs. 1 and 2) (Vogt and Pandya 1987; Petrides and Pandya 1999; Leichnetz 2001; Kobayashi and Amaral 2003, 2007; Vann et al. 2009; Milczarek and Vann 2020). The functional interaction between these areas was examined by means of a powerful method, namely the crossed unilateral lesion method (see Vaidya et al. 2019). A unilateral lesion within the mid-dorsolateral prefrontal cortex (areas 46 and 9/46) in one hemisphere was paired with a lesion in the medial parietal area PGm in the contralateral hemisphere (Figs. 2 and 4). Consistent with the prediction from the anatomical connectivity, this combination of lesions impaired the performance of monitoring information within working memory (Fig. 5). Thus, these results demonstrate for the first time the functional role of precuneal medial parietal area PGm in providing the *interaction* between the critical prefrontal region for the executive control process of monitoring information, the multisensory attentional system in lateral parietal area PG, and the hippocampal memory system.

The externally ordered nonspatial working memory task that assesses monitoring of information within working memory was used in this experiment (Petrides 1995). In this task, the monkeys are first shown sequentially a randomly selected subset of 2 stimuli from a familiar set of 3 stimuli (or 3 stimuli from a familiar set of 4 stimuli) and, on the subsequent test phase of the trial, they are faced with all the familiar stimuli and have to select the stimulus that had not been presented earlier on that particular trial (Fig. 3). Note that the location of these stimuli in the test phase of the trial varies randomly, making the use of spatial coding impossible. Thus, correct performance requires the online monitoring of the occurrence/non-occurrence of stimuli from a familiar set and is based entirely on working memory of the visual stimuli and not their locations. Bilateral selective lesions of the mid-dorsolateral prefrontal cortex impair performance on these nonspatial working memory tasks in the macaque monkey, but bilateral lesions of the adjacent dorsolateral prefrontal region that includes area 8A and dorsal area 6 do not impair performance (Petrides 1995, 2000). The present results from the crossed unilateral lesion method that disconnected the mid-dorsolateral prefrontal cortex from the medial parietal area PGm demonstrated the critical functional interaction of the prefrontal region with the precuneal medial parietal area PGm for the performance of this task.

Based on the above impairment and the anatomical connectivity of the critical areas, the functional interactions can be conceptualized as follows. The mid-dorsolateral prefrontal cortical region is interacting with the multisensory attentional region in the inferior parietal lobule for the online monitoring of information but, as selections are made across time during this online processing, the memory of the earlier selections must be integrated with the current processing of the information to avoid re-selecting the previous stimuli. The anatomical connectivity of the precuneal medial parietal area PGm suggested that it may be the critical node for the integration of these aspects of cognitive processing given its strong links with both the mid-dorsolateral prefrontal cortex and the inferior parietal lobule, as well as with the ventrally adjacent limbic retrosplenial/posterior cingulate cortex that provides major input to the hippocampal memory system (see Figs. 1 and 2). The crossed unilateral lesion methodology which examines the *functional* interaction between brain areas provided the strongest possible evidence that indeed area PGm is a critical integration node for the above processes.

How do we relate the present findings to the human brain? In an earlier investigation with patients, impaired performance was demonstrated on a working memory monitoring task after unilateral prefrontal or unilateral medial temporal lesions involving the hippocampus (Petrides and Milner 1982). Furthermore, the relevance of these findings for the human brain is clearly shown in a functional neuroimaging study in which the exact same paradigm was used, the Externally Ordered Working memory task with verbal stimuli (Petrides et al. 1993b). In the externally ordered working memory condition, the subjects heard, during scanning, a random sequence of the numbers from 1 to 10, with one number omitted in each presentation trial. The subjects had to monitor carefully on each trial the random presentation of the numbers because, on completion of the trial, they would have to report the number that had been omitted in that particular trial. In this condition, there was activation within the dorsolateral prefrontal cortex and the lateral parietal cortex (i.e. the fronto-parietal network) and, also, activation in the precuneal medial parietal area PGm, i.e. medial area 7 in the Brodmann terminology (Petrides et al. 1993b).

In future studies, the functional role of area PGm in interaction with other associated cortical areas can be further examined with additional methods. For example, the development of the optogenetic method permits investigators to perturb activity of specific neurons in a particular cortical region and examine the effects on behavior (e.g. Han et al. 2009). Similarly, chemogenetic approaches permit the manipulation of neuronal function in particular areas and pathways in freely moving animals (see Campbell and Marchant 2018, for a review of this methodology). Furthermore, electrophysiological recording from single neurons and groups of neurons within area PGm can provide important information to interpret its specific functional role (see Hong and Lieber 2019, for a review of this approach).

The role of the inferior parietal lobule is often interpreted in a narrow context, i.e. to imply processing of information about locations. There is no doubt that the lateral inferior parietal lobule provides the spatial context within which experience occurs (Behrmann et al. 2004; Shomstein and Gottlieb 2016). However, this important observation must not be misinterpreted as the processing of only locations. For example, Sereno and Maunsell observed visual shape selective neuron responses within the lateral intraparietal cortical region in macaque monkeys (Sereno and Maunsell 1998). Many other studies have shown that the inferior parietal lobule provides the attentional framework for the online processing of multisensory information (e.g. Gifford and Cohen 2005; Konen and Kastner 2008; Margulies et al. 2009; Shomstein and Gottlieb 2016). In the EOM working memory task, the monkey was required to monitor the occurrence/non-occurrence of visual stimuli and position was irrelevant, i.e. it could not be used for the coding of the stimuli because their position varied randomly throughout the task. The decision to use a strictly *nonspatial* working memory task was made so that the role of the precuneal medial parietal area PGm would be shown to underlie general nonspatial information processing in working memory. The powerful crossed unilateral lesion methodology used here demonstrated the critical role of the posterior medial parietal region (area PGm) in interaction with the lateral prefronto-parietal cortical network for the performance of this nonspatial working memory task.
